# Improving onchocerciasis elimination surveillance: trials of odour baited Esperanza Window Traps to collect black fly vectors and real-time qPCR detection of *Onchocerca volvulus* in black fly pools

**DOI:** 10.1186/s13071-024-06554-5

**Published:** 2024-11-18

**Authors:** Monsuru A. Adeleke, Kenneth N. Opara, Hayward B. Mafuyai, Bertram Ekejiuba Bright Nwoke, Olabanji A. Surakat, Sunday B. Akinde, Murphy Nwoke, Friday M. Chikezie, Clement A. Yaro, Ugagu Mmaduabuchi, Michael Igbe, Emeka Makata, Fatai Oyediran, Chukwuma Anyaike, Joseph Tongjura, Frances Hawkes, Zahra O. Iwalewa

**Affiliations:** 1 Department of Zoology, Osun State University, Osogbo, Nigeria; 2 Department of Animal and Environmental Biology, University of Uyo, Uyo, Nigeria; 3 Department of Zoology, University of Jos, Jos, Nigeria; 4 Department of Animal and Environmental Biology, Imo State University, Owerri, Nigeria; 5 Department of Microbiology, Osun State University, Osogbo, Nigeria; 6 Federal Ministry of Health, Abuja, Nigeria; 7 Department of Biological Science, Nassarawa State University, Lafia, Nigeria; 8 Agriculture, Health & Environment Department, Natural Resources Institute, University of Greenwich, London, UK

**Keywords:** *Simulium damnosum* s.l., 2-Butanone, Cyclopentanone, Carbon dioxide, Esperanza Window Trap, Real-time PCR, Onchocerciasis

## Abstract

**Background:**

Entomological data for onchocerciasis surveillance relies on sampling black flies through human landing collectors in the field and laboratory testing of the flies for infection using pooled screening O-150 PCR-ELISA assay. Both techniques require improvements. This study aimed to optimize the Esperanza Window Trap (EWT) for black fly collection. We tested alternative carbon dioxide (CO_2_) mimics to attract black flies to the traps. Additionally, we evaluated new quantitative PCR (qPCR) methods that target mitochondrial DNA markers and have been proposed to enhance the sensitivity and specificity for detecting *Onchocerca volvulus* infections in blackflies.

**Methods:**

Traps baited with low, medium and high release rates of either 2-butanone or cyclopentanone as CO_2_ mimics were field tested against traps baited with organically generated CO_2_ in four ecological zones in Nigeria: Guinea savannah, derived savannah, rainforest and montane forest. The performance of EWTs baited with CO_2_ or in combination with 2-butanone (low release) were subsequently evaluated against the human landing collection (HLC). Trap scaling was also pilot tested by comparing two EWTs to a single HLC team. Collected black flies were used to test detection of *O. volvulus* in black flies using Ov ND5 real-time PCR (qPCR) compared to the conventional pool screening O-150 PCR.

**Results:**

EWTs baited with 2-butanone caught similar numbers of black flies (*Simulium damnosum* s.l.) to those baited with CO_2_, while cyclopentanone collected significantly fewer flies in all locations. The low release of 2-butanone was the most effective overall, although HLCs collected higher numbers of black flies than EWT baited with CO_2_ either singly or in combination with low-release 2-butanone. The combination of two EWTs baited with CO_2_ and deployed 100 m apart from each other collected similar numbers of flies as one HLC. More black fly pools were positive for *O. volvulus* by Ov ND5 qPCR compared with O-150 PCR in derived savannah (31.15 vs. 15.57%), montane forest (11.54 vs. 0%) and rainforest (23.08 vs. 2.56%), with only one positive pool in Guinea savannah detected by both methods.

**Conclusions:**

The 2-butanone has potential to be used in xenomonitoring as a standardized replacement for organically generated CO_2_. Ov ND5 qPCR detected more positive pools than O-150 PCR. The positive pools found in foci hitherto considered to have interrupted/eliminated onchocerciasis highlight the need for more sensitive and specific methods that support programmatic assessments to identify and combat recrudescence.

**Graphical Abstract:**

**Figure:**
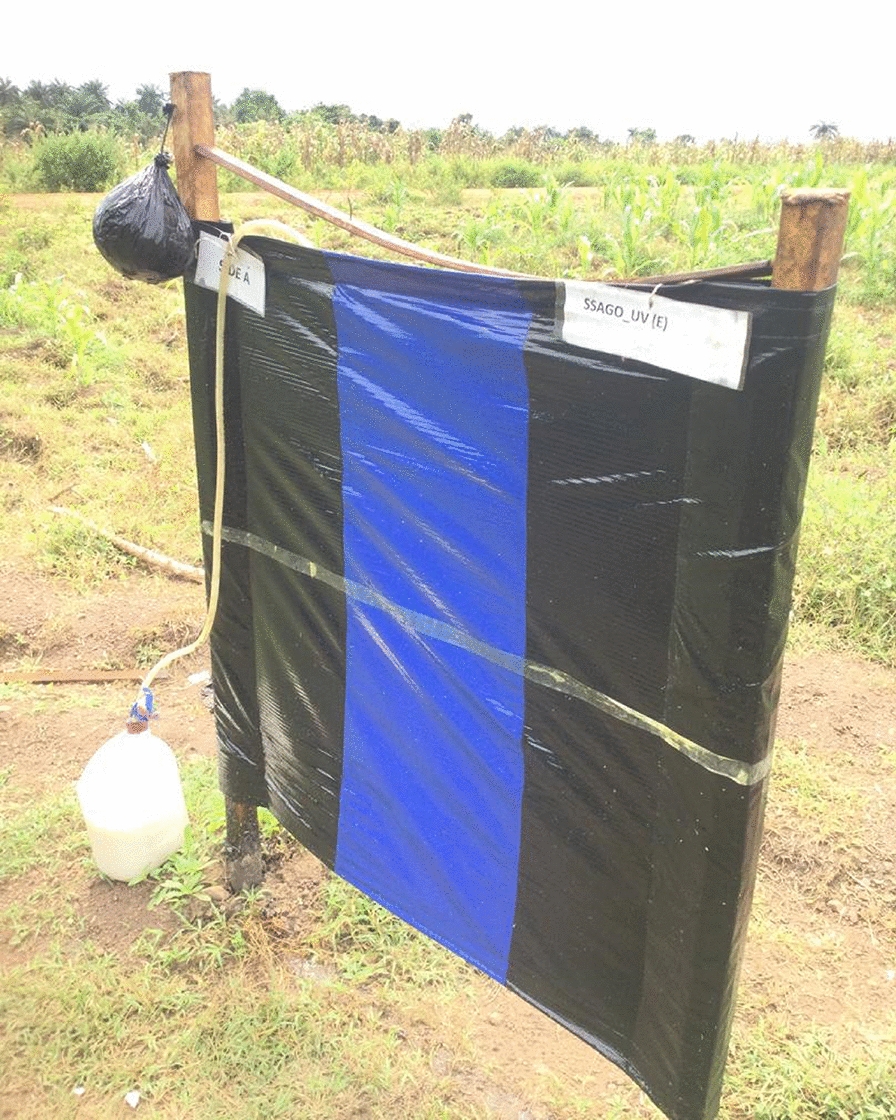

## Background

Human onchocerciasis (river blindness) is a debilitating disease of significant public health importance, occurring primarily in Africa, which bears the brunt of the infection [1]. The disease is known for its characteristic skin pigmentation, itching and blindness depending on the *Onchocerca volvulus* strain circulating in a transmission zone. The parasite is transmitted by blackflies of the genus *Simulium*, thus making the insect an indispensable indicator for real-time assessment of the transmission of the disease and for monitoring the impact of control interventions in endemic foci [2].

The control, and now elimination, of onchocerciasis has relied mostly on annual mass drug administration (MDA) of ivermectin with some complementary vector control strategies in a few countries [3]. It is expected that the long-term administration of ivermectin of between 12 and 15 years, with good coverage, has potential to interrupt the transmission of the disease to below the threshold at which recrudescence would not be possible [1]. Thus, WHO guidelines place utmost emphasis on epidemiological and entomological impact assessment to determine whether the drug can be safely stopped after long-term administration in the transmission zones. Entomological assessments rely on the field collection of black flies through human landing collection (HLC) technique and subsequent laboratory detection of *O. volvulus* using O-150 PCR-ELISA technique on pools of the heads of the flies [4]. However, several issues have been raised about these two techniques: the ethical appropriateness and cost of using humans as bait for black fly collection [5] and the sensitivity of the O-150 PCR-ELISA in detecting the true-positive samples in pools of black fly heads [6].

One of several efforts at developing an alternative trapping technique to replace HLC is the Esperanza Window Trap (EWT) [5]. These demonstrated great potential and reasonable logistical advantage for the collection of host-seeking African species of black flies [7]. It was suggested that one person can monitor multiple traps at a catching site and EWTs can be widely deployed for fly collection in the endemic communities [2]. However, the EWTs need further optimization for consistent performance, which can vary significantly across geographic zones as earlier reported [8, 9]. Previous studies have elaborated on the role of CO_2_ as an activating attractant to black flies [10, 11]. Simplifying and standardizing the source of CO_2_ to bait the EWT is one of the key improvements recommended for further optimization [8]. The EWT is usually baited with CO_2_ generated by yeast fermentation [5]; however, the type of sugar, yeast strain and environmental conditions affect the production rate of organically generated CO_2_ under field conditions [8]. A recent study on another haematophagous Dipteran revealed that traps baited singly with granules of cyclopentanone or in combination with other attractant blends collected more *Anopheles arabiensis* than those baited with yeast fermentation-produced CO_2_ [12]. Similar studies [13] reported significant attraction of *Anopheles gambiae* s.l. to 2-butanone compared to industrially supplied CO_2_. The potential application to collection of anthropophilic blackflies has not yet been explored.

Once collected, black fly samples must be screened in the laboratory to determine whether they harbour infective (L3-stage) larvae. The current WHO-endorsed molecular technique targets the O-150 repeat for amplification [14], but the reagents require a reliable cold chain which cannot always be guaranteed during shipment or where electrical power supplies are inconsistent. A recently developed real-time qPCR method using Ov ND5, a mitochondrial DNA target, uses heat-stable reagents and is cheaper and faster to complete than the O-150, while also demonstrating increased sensitivity and specificity in initial comparisons [15]. This warrants further field evaluation to determine its suitability for use in programmatic settings.

It is against this background of operational roadblocks to verifying onchocerciasis elimination that the present study sought to (i) evaluate and compare the efficiency of different release rates of cyclopentanone and 2-butanone (CO_2_ mimics) as attractants for black fly vectors of onchocerciasis, (ii) evaluate the synergistic potential of EWTs baited with a combination of CO_2_ mimic and yeast-fermentation generated CO_2_ compared to HLCs and (iii) evaluate the detection of *O. volvulus* in head pools of the collected black flies using O-150 PCR-ELISA versus Ov ND5 qPCR assays.

## Methods

### Study area

**Fig. 1:**
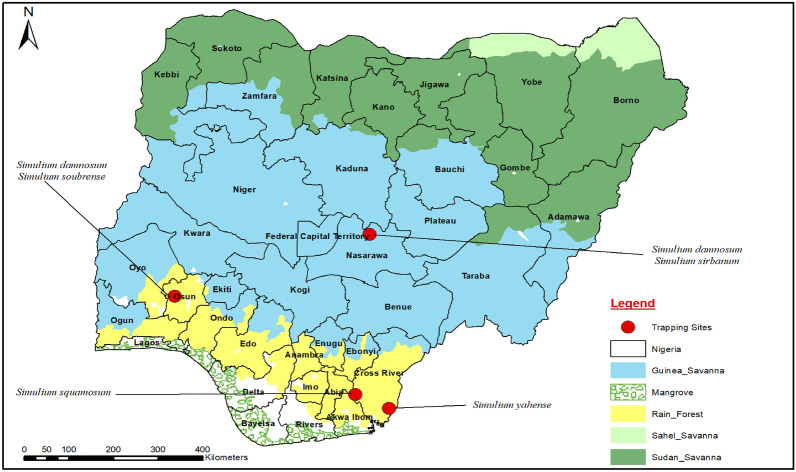
Map of Nigeria showing the study locations and the ecological zones (Arc GIS software version 10.7.1, ESR available at https://www.R-project.org/ was used to produce the map while the Nigerian shapefile was obtained from World Bank Data atalogue—an Open license standardized resource of boundaries for every country in the world)

Four onchocerciasis endemic locations representing four ecological zones with varying cytospecies of blackflies in the *Simulium damnosum* s.l. present were selected based on previous studies [16–20]. At each of the selected locations, two trap sites (at least 5–10 km apart) were established along the major river. The selected locations are described in Fig. 1 and Table 1. Field experiments were conducted between September and October 2023, coinciding with the high biting rate period of blackflies in the area.

**Table 1:** Study location characteristics and coordinates of the selected points

Ecological regions	Study Locations/States	Latitude/Longitude of trap sites	Previously identified black fly species	Onchocerciasis elimination status
Guinea savannah	Ungwan Habu, Nasarawa State	Trap site 1: 8.944399N, 8.242172E Trap site 2: 8.996879N, 8.256494E	*Simulium damnosum* s.s *S. sirbanum*	Interrupted [32, 33]
Montane Forest	Kwa Falls and Ekong Anaku, Cross River State	Trap site 1: 5.105748N, 8.630302E Trap site 2: 5.141645N, 8.508362E	*S. yahense* *S. squamosum*	On track to elimination [33]
Rain forest	Ike-Isu, Abia State	Trap site 1: 5.41310N, 7.95398E Trap site 2: 5.416057N, 7.954063E	*S. squamosum*	Interrupted [33]
Transition zone (Forest/derived savannah)	Ileogbo, Osun State	Trap site 1: 7.580873N, 4.301190E Trap site 2: 7.587072N, 4.289398E	S. *soubrense* Beffa *S. damnosum* s.s	On track to elimination [33]

### Field trials to improve black fly catches in EWTs

The field trials were divided into three experiments for optimization of EWTs. Experiments 1 and 2 concerned enhancing the olfactory attractivity of EWTs by testing CO_2_ mimics and augmenting organically produced CO_2_, and Experiment 3 tested the potential to increase overall black fly yield through increasing EWT sampling effort by comparing the catches of two EWTs deployed strategically within 100 m apart versus two individuals (working alternately) as human landing collectors. Experiments 1 and 2 were conducted at locations 1 and 2 in each of the ecological zones while Experiment 3 was conducted at location 2 in the derived savannah upon completion of experiments 1 and 2.

Carbon dioxide and its mimics used in Experiment 1 and 2 were produced as follows: 2-butanone and cyclopentanone were procured from Chemitica Inc., Costa Rica. Each of the two CO_2_ mimics was prepared in semipermeable membrane sachets in three slow-release modes, all of which were formulated for release at temperatures of 20–30 °C as follows: low release (~ 1–2 mg/day), medium release (~ 5–10 mg/day) and high release (~ 45–55 mg/day). The slow release enables the blend to mimic natural concentrations [21], last longer and not be repellent. Most of the semio-chemicals used for attracting insect vectors are slow release and can attract vectors over 30–100 m away [21]. The organically generated CO_2_ was prepared on alternate days (every 2 days) mixing 50 g dry baker's yeast, 500 g sugar and 2.5 l water in a 5-l plastic bottle at least 2 h before the commencement of trapping as earlier described by [8].

### *Experiment 1—identification of optimum CO*_*2*_* mimic release rates*

**Fig. 2:**
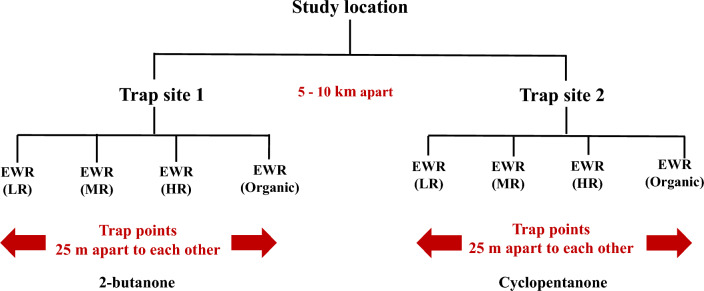
Schematic representation of the study design (*LR* low release, *MR* medium release, *HR* high release)

**Fig. 3:**
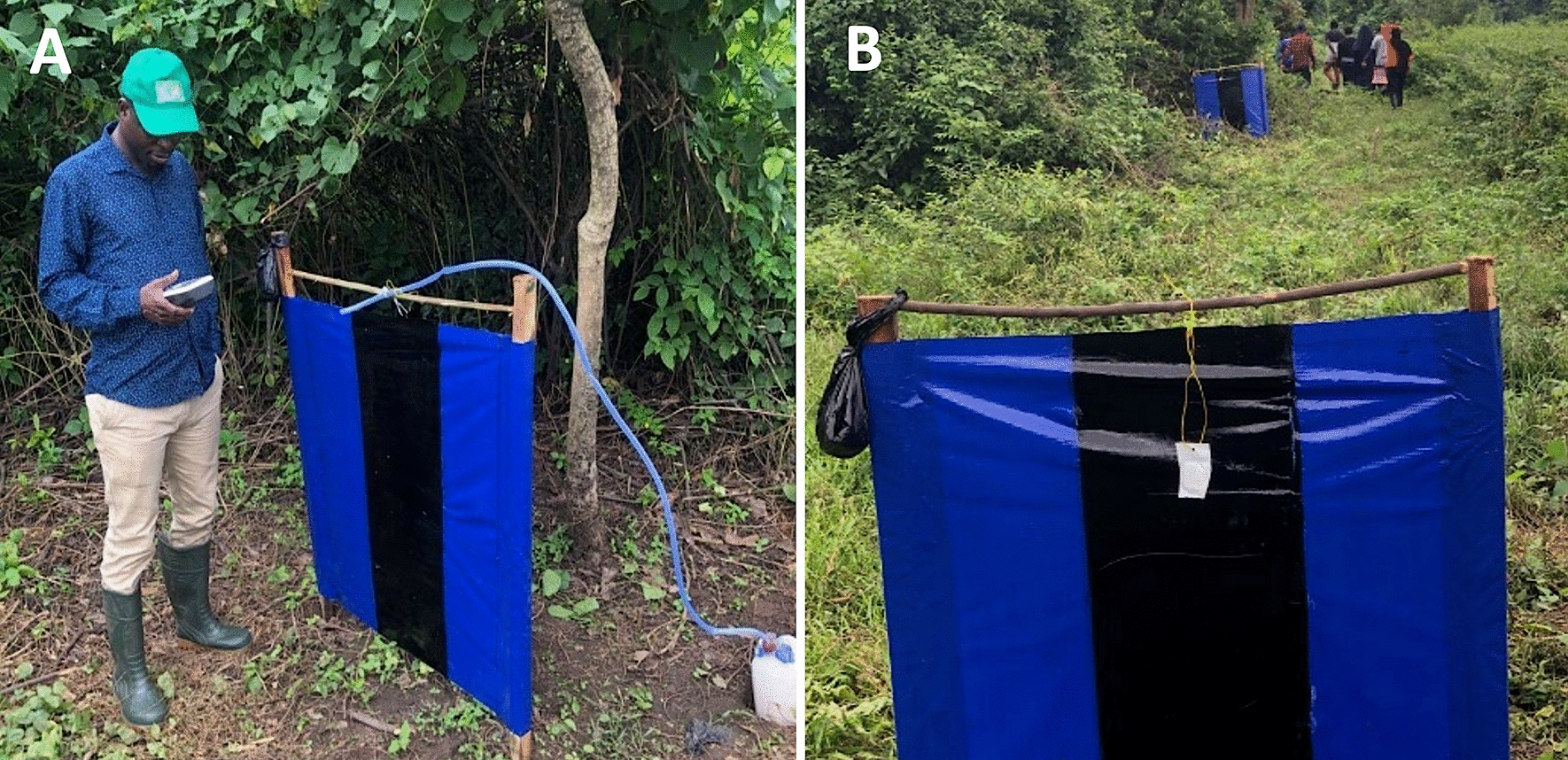
Esperanza Window Traps baited with (**A**) organically generated Carbon dioxide (circled in red) and (**B**) CO_2_ mimic (circled in red)

Two trapping sites, at least 5 km apart and known close to black fly breeding sites, were selected along the river in each of the four study locations (Table 1). Of these, 2-butanone was tested at the first trapping site (Trap site 1), while cyclopentanone was tested at the second (Trap site 2). At each trap site, four points (25 m apart) were selected for the placement of traps: the EWTs comprised a tarpaulin (1 × 1 m) with blue-black-blue stripes of equal size hung on an upright stand of poles tensioned with string and pegs and suspended about 10 cm above the ground. The two sides of the tarpaulin were coated with TAD^™^ All Weather (adhesive) (Ladd Research Industries, USA) and baited with a pair of worn socks hung at the top centre of the trap [7, 8]. The EWTs at the four points were also baited with low, medium or high release rate CO_2_ mimic or organically generated CO_2_ (Figs. 2 and 3). The traps were set up perpendicular to the breeding site close to a partly shaded tree. The traps were rotated daily in a randomized Latin square experimental design over a 12-day period to avoid position bias. Flies were recovered from the traps twice daily (12 noon and 5 p.m.) by removing all the flies caught by the traps into a tray containing motor spirit/kerosine using flexible forceps/a sharp toothpick. The glue was washed off the flies and the black flies caught were preserved with 80% ethanol in 30-ml bottles and kept at 4 °C until later use.

### Experiment 2—optimization of EWTs through augmented odours relative to HLC

This experiment was designed to determine whether the number of flies attracted to organically generated CO_2_ could be increased by adding a CO_2_ mimic. For 8 consecutive days at each study location, catches from an EWT baited with the standard organically generated CO_2_ were compared with an HLC positioned 25 m away from the trap. The HLC involved two adults collecting black flies on hourly rotation between 7 a.m. and 5 p.m. daily in accordance with the standard procedure [8, 9, 22]. The positions of the HLC and EWT were rotated daily in a cross-over design. On the 9th day, the organically generated carbon dioxide at the EWT was augmented with the best CO_2_ mimic from Phase 1, identified as 2-butanone at a low release rate, and continued to be compared with the HLC in a cross-over design until day 16. The daily catches were recovered from the traps as described in Phase 1.

### Experiment 3—trap scaling pilot

As trapping approaches offer the advantage of allowing multiple traps to be operated by a single field entomologist, but have the disadvantage of collecting fewer flies, the aim of this experiment was to establish in principle whether doubling the number of EWTs baited with organically generated CO_2_ would increase total trap catch relative to an HLC. This phase was only conducted at Ileogbo, the derived savannah zone in Osun State. Two EWTs were placed 100 m apart from each other and from the HLC position. The trial was conducted over 9 days and positions were rotated daily, following the collection and sampling methods described above. The number of flies collected daily by the two traps were added together and compared with the HLC catches.

### Fly infectivity in pools by O-150 PCR ELISA vs. Ov ND5 real-time qPCR

All the flies collected in the methods described above were identified morphologically for confirmation of female *S. damnosum* complex using the standard keys [4]. The heads of the flies were separated from the body using entomological pins under the dissecting microscope (Biobase). The batches of the pins used were washed with sodium hypochlorite and sterilized before being re-used to minimize contamination. The heads were arranged in 100 heads per pool and each pool was transferred to a sterilized Eppendorf tube for DNA extraction. The extraction of the DNA was done using Qiagen DNeasy Blood & Tissue Kits (Qiagen, Germany) following the manufacturer’s instruction. The O-150 PCR and Ov ND5 gene real-time qPCR assays for the detection of *O. volvulus* in the pools were performed as previously described by [2, 23].

### Statistical analysis

The data on blackfly count from the various collection methods were analysed using a generalised linear mixed model (GLMM) with a negative binomial distribution and counts were compared across release rates. Means were then separated using *post hoc* Tukey’s multiple comparison test. A generalised linear model (GLM) was used to compare the means of daily catches between EWT and HLC. The prevalence of infection in the pool screening of fly heads was determined using Poolscreen v2.0 software [24]. All other analysis was performed using statistical package R-4.3.0 [25].

## Results

### Field trials to improve blackfly catches in EWTs

#### Experiment 1: identification of optimum CO_2_ mimic release rates

**Fig. 4:**
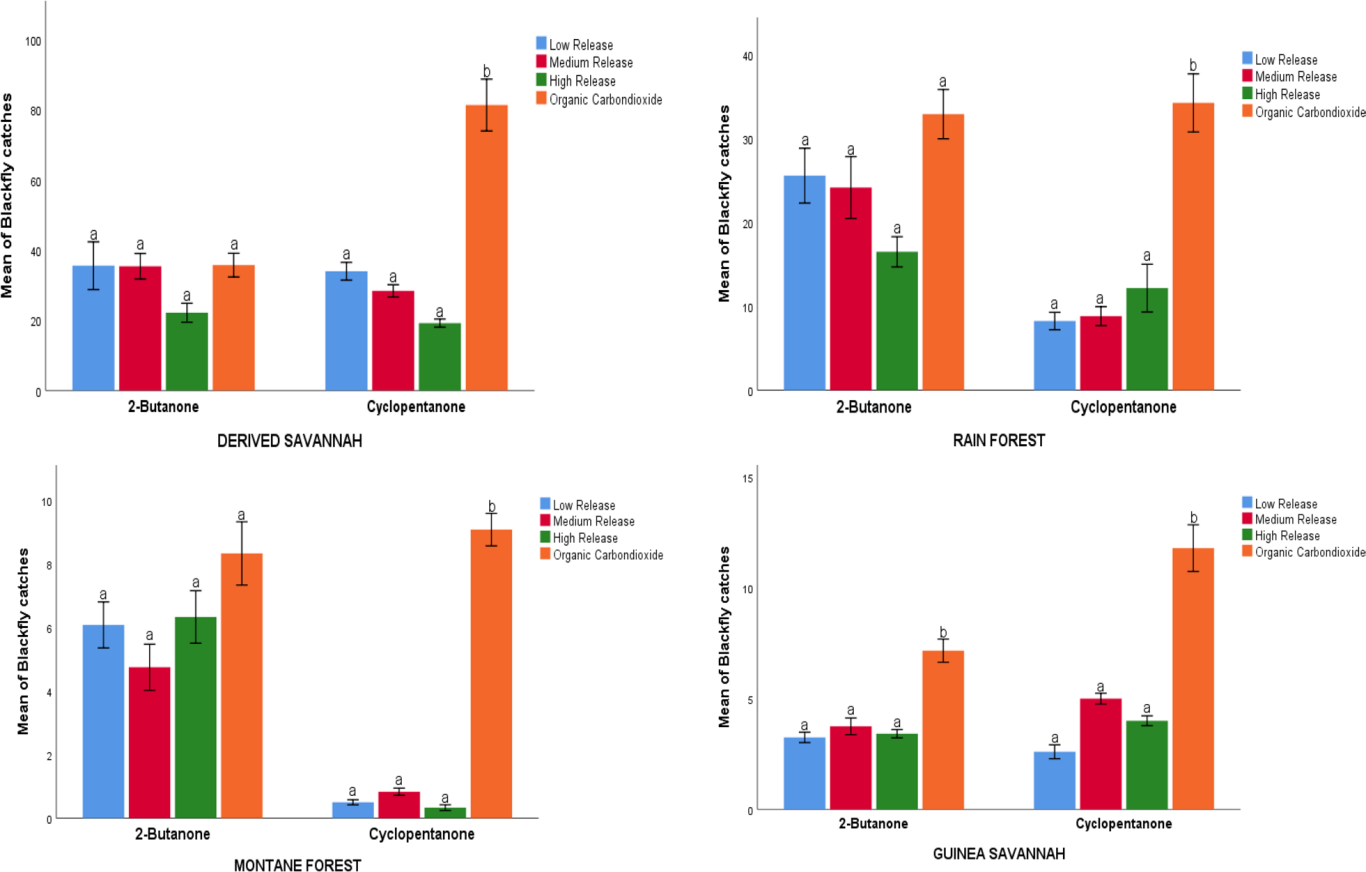
Mean daily black fly catches (± standard error) of EWTs baited with different release rates of CO_2_ mimics and organically produced CO_2_ in four different ecoregions of Nigeria. Different letters denote significant differences at *p* < 0.05 (GLM)

The results obtained from the optimization of 2-butanone and cyclopentanone against organically generated CO_2_ in the four study locations are presented in Fig. 4. In three of the four study areas, all release rates of 2-butanone performed well in terms of mean daily fly catch relative to the organically produced CO_2_. In the derived savannah (Fig. 4b), the mean catches of low release (35.58 ± 19.660), medium release (35.41 ± 10.542) and high release (22.16 ± 7.797) rates of 2-butanone were not significantly different from the mean catch by EWT baited with organically generated CO_2_ (35.75 ± 9.788) (*p* = 0.50). Likewise, in montane forest, mean catches from the low (6.08 ± 2.09), medium (4.75 ± 2.09) and high (6.33 ± 2.38) release rates did not differ significantly compared to that from organic CO_2_ (8.33 ± 2.87) (*p* = 0.833), and this was also the case in the rain forest ecoregion, where low (25.58 ± 9.44), medium (24.16 ± 10.63) and high (16.50 ± 5.23) release rates did not yield significantly more black flies per day than organic CO_2_ (32.91 ± 8.40); *p* = 0.409). However, in Guinea savannah, organically produced CO_2_ caught significantly more flies per day (7.16 ± 1.05) than any of the 2-butanone-baited traps (low 3.25 ± 0.67, *p* = 0.046; medium 3.75 ± 1.08, *p* = 0.049; high 3.41 ± 0.54, *p* = 0.048).

In contrast, cyclopentanone performed poorly across all ecoregions. The EWT baited with organic CO_2_ in derived savannah zone collected a significantly higher mean number of black flies per day (81.40 ± 36.987) than EWT baited with cyclopentanone for low release (34.00 ± 12.677; *p* = 0.047), medium release (28.40 ± 8.704; *p* = 0.049) and high release (19.20 ± 5.774; *p* = 0.048). Similar results were obtained in rainforest (organic CO_2_: 34.25 ± 9.99; low release: 8.25 ± 2.98, *p* = 0.048; medium release: 8.83 ± 3.26, *p* = 0.048; high release: 12.16 ± 8.22, *p* = 0.046); derived savannah (organic CO_2_: 11.80 ± 4.71; low release: 2.66 ± 1.40, *p* = 0.046; medium release: 5.00 ± 1.09, *p* = 0.049; high release: 4.00 ± 1.00, *p* = 0.048); montane forest (organic CO_2_: 9.08 ± 1.47; low release: 0.50 ± 0.23, *p* = 0.000; medium release: 0.83 ± 0.32, *p* = 0.002; high release: 0.33 ± 0.28 *p* = 0.000).

### Experiment 2: optimization of EWTs through augmented odours relative to HLC

**Fig. 5:**
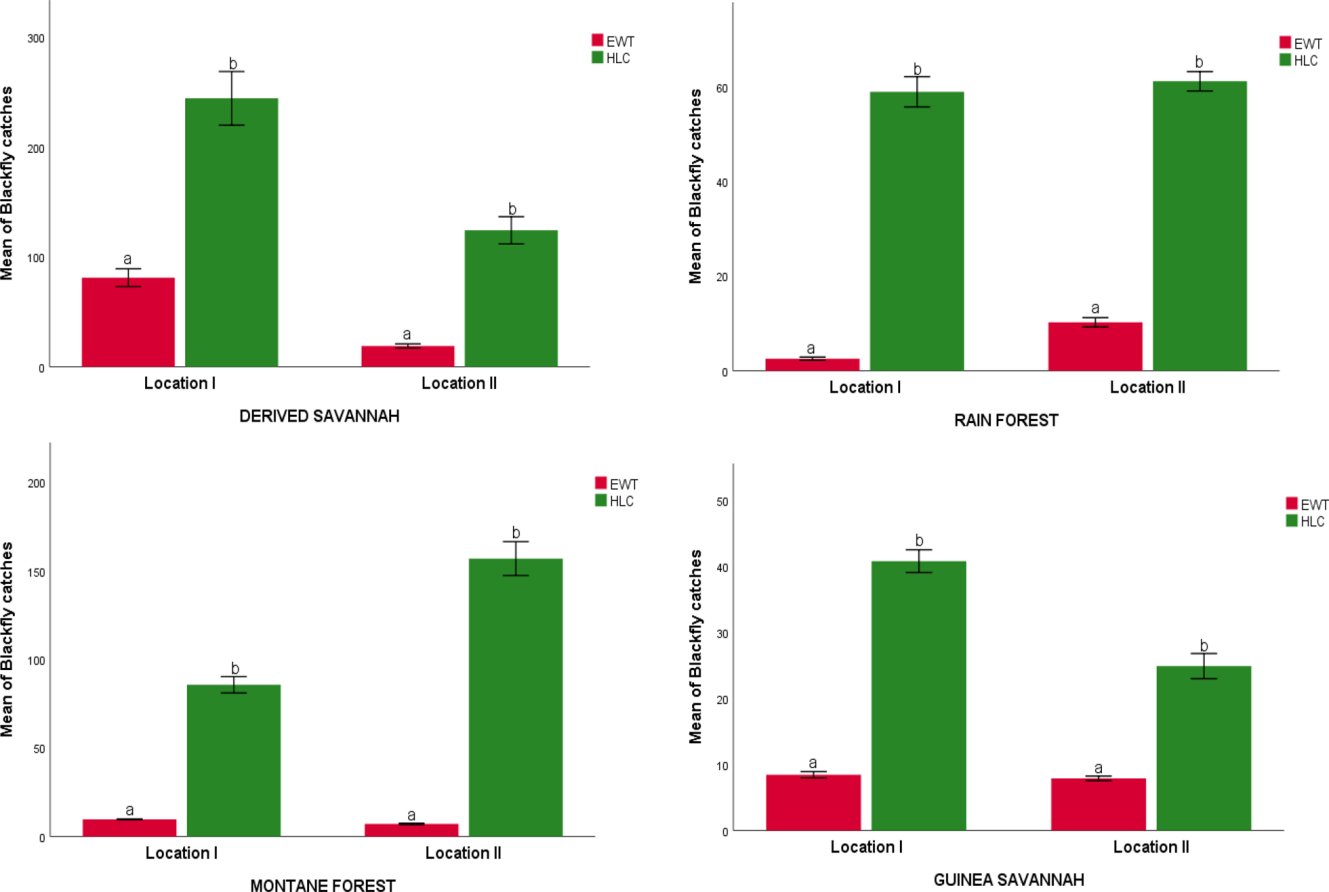
Mean daily black fly catches (± standard error) by EWTs baited with organic CO_2_ versus human landing collection in four different ecoregions of Nigeria. Different letters denote significant differences at *p* < 0.05 (GLM)

**Fig. 6:**
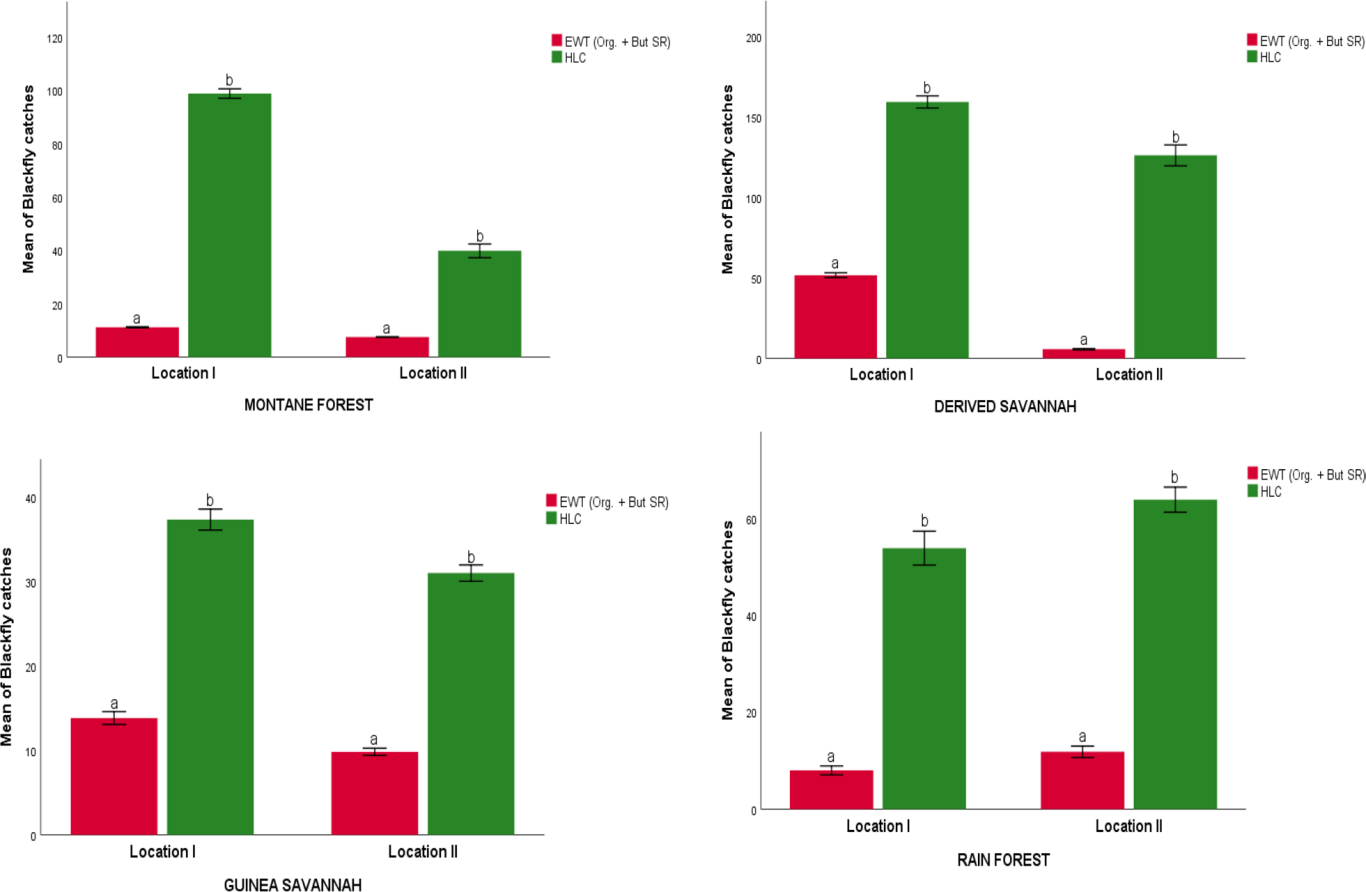
Mean daily black fly catches (± standard error) by EWTs (baited with combination of 2-butanone low release rate plus organic CO_2_) versus human landing collection in four different ecoregions of Nigeria. Different letters denote significant differences at *p* < 0.05 (GLM)

HLC collected a significantly higher number of black flies per day than EWTs baited with organically generated CO_2_ across the locations (*p* < 0.05). In location I in the four ecozones, for example, the mean catches by HLC versus EWT were given as derived savannah = 244.11 ± 22.063 vs. 81.000 ± 9.402; rainforest = 58.889 ± 10.671 vs. 2.556 ± 1.107; montane forest = 85.667 ± 15.304 vs. 9.667 vs. ± 0.782; Guinea savannah = 40.778 ± 5.746 vs. 8.444 ± 1.591 (Fig. 5). The EWTs baited with the combination of 2-butanone (low release) and organically generated CO_2_ also collected significantly fewer black flies than HLC across the study locations (*p* < 0.05) (Figs. 5 and 6).

### Experiment 3: trap scaling pilot

**Fig. 7:**
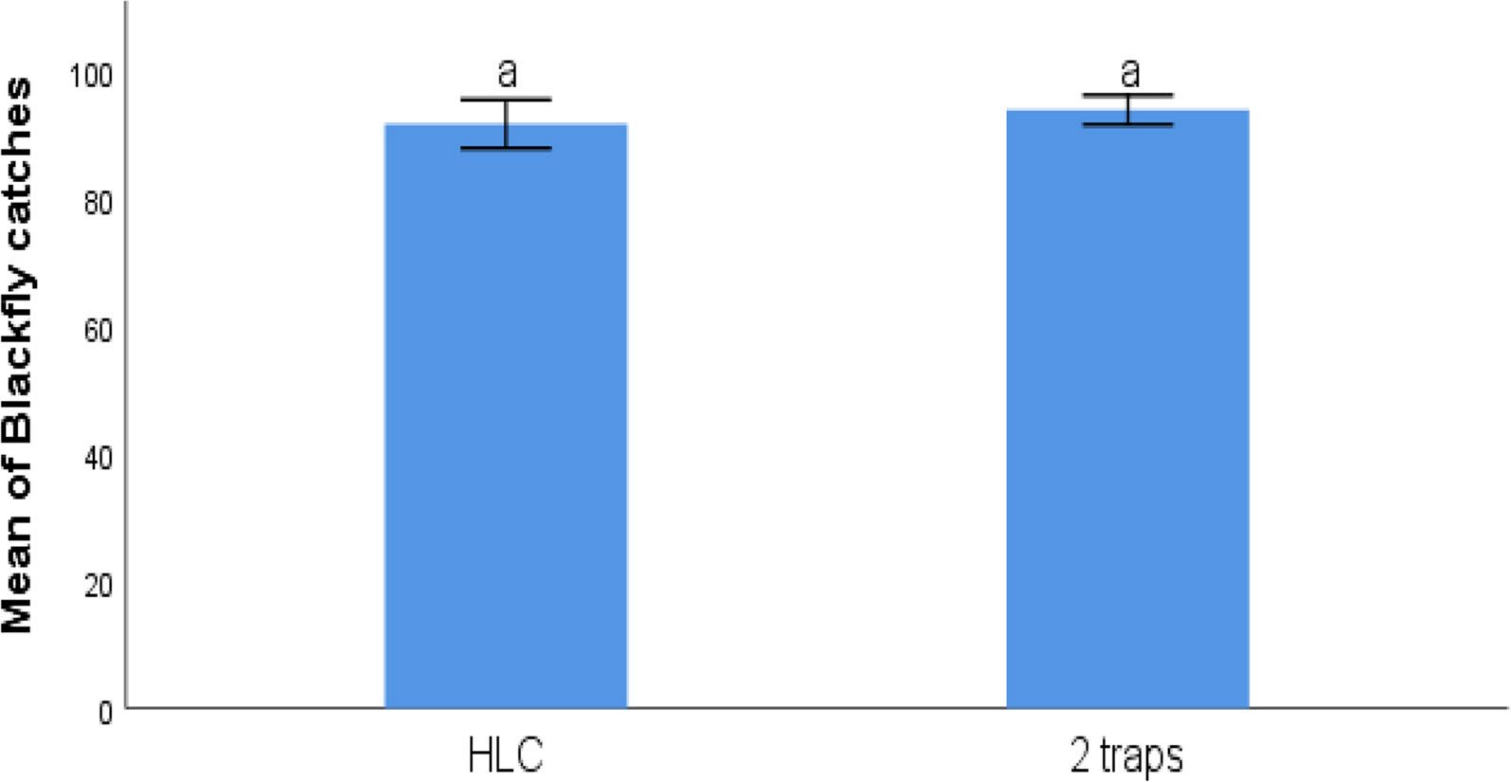
Mean daily black fly catches (± standard error) by two EWTs (baited with organic CO_2_ 100 m apart) versus human landing collection (100 m apart to the trap) at Ileogbo in Osun State, Nigeria, a location in derived savannah. Different letters denote significant differences at *p* < 0.05 (GLM)

The combination of two EWTs (each baited with organic CO_2_ and deployed 100 m apart from each other and from the HLC) collected slightly more flies than the HLC. The pooled mean catch of both traps was 94.011 ± 5.97, which was very similar to the HLC catch of 91.80 ± 9.86, a difference that was not statistically significant (*p* = 0.788) (Fig. 7).

### Fly infectivity in pools by O-150 PCR ELISA versus Ov ND5 real-time qPCR

The results of the molecular analyses of black fly infectivity showed that all the study locations had at least one positive pool by both O-150 PCR and Ov ND5 qPCR, with the exception of montane forest, which was positive only by Ov ND5 qPCR (Table 2). More positive pools were detected by Ov ND5 qPCR compared to O-150 PCR in three of the four ecoregions: in Guinea savannah (31.15 vs. 15.57%), montane forest (11.54 vs. 0%) and rainforest (23.08 vs. 2.56%). Only one pool was positive by both Ov ND5 qPCR and O-150 PCR in Guinea savannah. All pools that were positive by O-150 PCR were also positive by Ov ND5 qPCR. The upper limit prevalence was exceedingly higher than 0.05% in all four locations using both diagnostic methods except at montane forest, which has a lower value for O-150 PCR (Table 2).

**Table 2:** Prevalence of *Onchocerca volvulus* in black flies at four study locations in Nigeria

Ecoregions (states)	No. of pools	No. of pools positive by O-150 PCR (%)	No. of pools positive by Ov ND5 qPCR (%)	Prevalence by O-150 PCR (95% confidence interval)	Prevalence by Ov ND5 qPCR (95% confidence interval)
Derived savannah (Osun)	122	19 (15.57)	38 (31.15)	0.16 (0.10–0.26)	0.37 (0.29–0.51)
Montane forest (Cross River)	78	0 (0)	9 (11.54)	0.00 (0.00)	0.12 (0.06–0.22)
Rainforest (Abia)	39	1 (2.56)	9 (23.08)	0.03 (0.00–0.14)	0.26 (0.14–0.48)
Guinea savannah (Nasarawa)	06	1 (16.67)	1 (16.67)	0.18 (0.03–0.82)	0.18 (0.03–0.82)

## Discussion

Carbon dioxide serves as a powerful attractant to haematophagous insects, and it has been widely used for traps to lure host-seeking insect vectors [10, 11, 13, 26]. In a search to improve the efficiency and easy deployment of EWTs, our field evaluation of various release rates of 2-butanone and cyclopentanone showed that the catches of EWTs baited with 2-butanone were not statistically different from those baited with organic CO_2_ across the study locations except at Guinea savannah, where significantly lower catches were recorded for 2-butanone. The reason for this is not clear; however, it may be species-specific as, unlike other study areas, this Guinea savannah location was dominated by *S. damnosum* s.s. and *Simulium sirbanum*. The lowest release rate of 1–2 mg/day was sufficient to catch similar numbers of blackflies as the medium and high release rates tested. Turner et al. [27] reported dose-dependent activation of receptor neurons in the maxillary palps of mosquitoes to 2-butanone; this has not been explored in black flies and our field results did not indicate a dose effect so far as the release rate did not significantly affect trap capture rates. 2-Butanone is a known natural product emanated by various vertebrates with potential to serve as a long-range attractant to host-seeking insects mimicking the CO_2_ produced by vertebrate hosts, including humans [13]. These properties could have accounted for its effectiveness in collecting flies almost at par with EWTs baited with organically generated CO_2_ but not exceeding these. Fermentation of sugar by yeast has been reported to produce not only CO_2_ but also other volatile organic compounds, including alcohols, esters, aldehydes and fatty acids, which also attract arthropods [5, 28] and may explain its slightly greater success as a lure.

However, EWTs baited with cyclopentanone collected significantly fewer black flies than those baited with organically generated CO_2_ across the four locations. Two reasons were advanced to explain the poor performance of cyclopentanone in the field: (i) the diffusion of odour plumes of the chemical due to environmental factors such as relative humidity, temperature and wind and (ii) competing chemicals emanating from the surrounding vegetation, detritus and air pollution in the field. The reason may be explained by the fact that cyclopentanone is a monoketone obtainable in plants and has been used widely in pharmaceuticals and industrial processes including in rubber chemicals [26, 29, 30]. Considering the typical ecological habitat of black flies, which is usually populated with shrubs and vegetation, the competing odour of this chemical from the surrounding vegetation is inevitable.

The EWTs baited with organic CO_2_ collected significantly fewer black flies to HLC across the four ecological zones. The reasons for differential trap performance are not clear. In the present study, augmentation of organic CO_2_ with low release rates of 2-butanone did not show any additional benefit in terms of catch, whereas in a field study conducted against *Anopheles* mosquitoes [13], augmentation of CO_2_ sources with 2-butanone significantly improved the catches of the mosquitoes. Similar observations have also been reported in Tanzania [8] and Cameroon [9]. In contrast, studies in Burkina Faso [7] and Uganda [8] reported a statistically similar number of flies collection between EWTs and HLC. Interestingly, when two EWTs baited with organic CO_2_ were used at a site in the derived savannah, collectively the two traps caught slightly higher numbers of black flies than two human landing collectors working rotationally on hourly basis. This result may be due to the fact that all the collection methods were well spread out, with approximately 100 m between the three catch points (EWT, HLC and EWT). The strong selection pressure on blackflies to respond to human-specific cues, and thus the highly attractive profile of human collections, may therefore be outcompeting alternative collection methods that only present partial and artificial attractive cues. This result needs further investigation in multiple sites in different ecological zones and countries against several cytospecies of *S. damnosum* s.l. This will help for standardization of EWT compared with the HLC (gold standard) in estimating the biting rate against cytospecies in different ecological contexts for alignment in programmatic use. Rodriguez-Perez et al*.* [2] demonstrated the field feasibility of deployment of multiple EWTs for the collection of the required numbers of flies to meet WHO guidelines requirement and our study reinforces this observation, and rigorous investigation in areas with different biting rates and cytospecies should be included as part of future studies.

The higher number of black fly head pools positive for Ov ND5 qPCR compared with O-150 PCR ELISA showed that the former is more sensitive than the latter. Notably, all the samples positive for O-150 PCR were also positive for Ov ND5 qPCR but the qPCR detected 36 more positive pools that had been scored negative by O-150 PCR. This result is in tandem with the recent studies commissioned by WHO [6] and published laboratory reports [15] on the high sensitivity of Ov ND5 qPCR over O-150 qPCR and O-150 PCR ELISA. Importantly, the Ov ND5 qPCR protocol is also faster, more cost effective and easier to use, requiring fewer reagents than the O-150 PCR ELISA. Alongside the growing number of similar reports, our observation has serious implications for the on-going global efforts in onchocerciasis elimination, as accurate detection of infected flies is crucial to informing safe stop-MDA decisions and robust post-elimination surveillance. Flies serve as a link between parasite and host, and they give a real-time indication on whether or not transmission has been interrupted/eliminated in a community [5]. Erroneous stoppage of MDA in areas with low but existing residual transmission could bring early recrudescence and jeopardize the gains achieved over many years of MDA. Further optimization of qPCR for wide implementation for impact assessment and post-elimination surveillance must be vigorously pursued if the global effort to accelerate eliminating onchocerciasis in endemic communities is to be achieved by the year 2030.

Notwithstanding the different diagnostic methods used, the results of the pooled screening in the four study zones are of significant interest to onchocerciasis elimination efforts in Nigeria. The results obtained in derived savannah (Osun State) and the montane forest (Cross River) showed that there is ongoing transmission in these foci; both zones remain under annual ivermectin coverage, which our results confirm is still required. The National Onchocerciasis Elimination Programme (NOEP) needs to aggressively improve the therapeutic coverage in the communities, as this entomological evidence suggests the presence of people harbouring active adult parasite worms at the study locations. However, the observation of positive pools in rainforest (Abia State) and Guinea savannah (Nasarawa State) is a surprise overlaid with serious implications for the ongoing efforts at eliminating onchocerciasis in Nigeria.

According to the NOEP [31] and the available literature [32, 33], Nasarawa State is one of the two states where onchocerciasis has been eliminated in Nigeria while Abia State has successfully interrupted the transmission of the disease based on the results of epidemiological and entomological assessments for stop-MDA assessment [31–33]. The occurrence of the positive pools at these two zones could be attributed to two possibilities: (i) that O-150 was not sensitive enough to detect the residual flies carrying infection after the MDA has suppressed active transmission since our study sites were among the communities where the Stop-MDA and Post Stop-MDA evaluations were conducted or (ii) the human migration caused by insecurity in Nasarawa and Abia States in the last 6 years has introduced sufficient numbers of infected people to the area to cause reinfection in the vector population. Irrespective of driving factors, the pool screening results have shown the possibility of a gradual build-up of recrudescence which requires urgent programmatic evaluation across Nasarawa and Abia States to determine the extent of the spread and instigate implementation strategies to combat it before it erodes the success already recorded in these transmission zones in Nigeria. To the best of our knowledge and available reports, this is the first report of direct comparison of O-150 PCR and Ov ND5 qPCR for the detection of blackfly infectivity in areas with ongoing transmission and in foci where onchocerciasis has been deemed interrupted/eliminated in West Africa.

## Conclusions

In conclusion, our study provides important findings for both vector collection and parasite detection within the context of the WHO’s onchocerciasis elimination framework. Our key results indicate that alternative odour baits and using multiple traps could play a role in securing the large number of black fly vector samples required for infection screening without relying on human collectors and that the Ov ND5 qPCR technique is a more sensitive method for detecting parasite DNA in these samples than the currently used O-150 method. Whether the positive pools found in the foci hitherto thought to have interrupted/eliminated onchocerciasis are caused by poor sensitivity of O-150 PCR ELISA or due to changing epidemiological factors, such as human migration due to insecurity, requires further programmatic evaluation by the NOEP in Nigeria.

## Data Availability

The data set for this research is available upon reasonable request from the corresponding author.

## Abbreviations

CO_2_: Carbon dioxide
DNA: Deoxyribonucleic acid
ELISA: Enzyme-linked immunosorbent assay
EWTs: Esperanza Window Traps
GLMM: Generalised linear mixed model
HLCs: Human landing collections
HR: High release
LR: Low release
MDA: Mass drug administration
MR: Medium release
NOEP: National onchocerciasis elimination programme
qPCR: Quantitative polymerase chain reaction
